# Supplementary material for: Pseudolycoriella hygida (Sauaia and Alves)—An Overview of a Model Organism in Genetics, with New Aspects in Morphology and Systematics
Source: Insects. 2024 Feb 6;15(2):118. doi: 10.3390/insects15020118 (PMC10889529; doi:10.3390/insects15020118)
Supplement: Supplementary file 1 [file insects-15-00118-s001.zip › insects-2831370-supplementary.pdf]

Table S1. Genetic distances (p-distances) based on COI barcodes from 14 outgroup representatives and 48 *Pseudolycoriella* specimens in %. The taxa have been arranged alphabetically within the two groups. *Pseudolycoriella hygida* specimens are marked in red.

	Species	1	2	3	4	5	6	7	8	9	10	11	12	13	14	15	16	17	18	19	20	21	22	23	24	25	26	27	28	29	30	31	32	33	34	35	36	37	38	39	40	41	42	43	44	45	46	47	48	49	50	51	52	53	54	55	56	57	58	59	60	61	62
1	***Bradysiopsis vittata*** GER BIOUG16734-E02																																																														
2	***Camptochaeta camptochaeta*** FIN BIOUG04085-F06	0.1155																																																													
3	***Claustropyga clausa*** NOR bf-sci-00255b	0.1292	0.1282																																																											
4	***Diadocidia ferruginosa*** GER BC-ZSM-DIP-24049-G09	0.1906	0.1888	0.1923																																																									
5	***Dichopygina nigrohalteralis*** NOR bf-sci-00193	0.1520	0.1630	0.1565	0.1958																																																							
6	***Hemineurina conspicua*** (conspicua group) NOR bf-sci-01103	0.1155	0.1424	0.1413	0.1871	0.1581																																																							
7	***Hemineurina inflata*** (inflata group) NOR bf-sci-01309	0.1444	0.1456	0.1535	0.2063	0.1657	0.1383																																																					
8	***Lycoriella sativae*** NOR TRD-Sci038	0.1550	0.1456	0.1535	0.1941	0.1581	0.1581	0.1733																																																				
9	***Protoxylosciara longiforceps*** NOR bf-sci-00889	0.1429	0.1709	0.1657	0.2185	0.1915	0.1611	0.1748	0.1854																																																		
10	***Spinopygina acerfalx*** USA JSSCI80	0.1383	0.1424	0.1292	0.1941	0.1626	0.1353	0.1702	0.1763	0.1581																																																	
11	***Stenacanthella freyi*** (freyi group) NOR bf-sci-01103	0.1474	0.1392	0.1398	0.1678	0.1733	0.1231	0.1413	0.1626	0.1596	0.1535																																																
12	***Stenacanthella secundaria*** (secundaria group) CAN BIOUG20457-G07	0.1313	0.1431	0.1414	0.2133	0.1717	0.1481	0.1431	0.1650	0.1380	0.1515	0.1498																																															
13	***Trichocoelina vitticollis*** NOR SV967	0.1550	0.1345	0.1489	0.2150	0.1611	0.1444	0.1520	0.1474	0.1824	0.1550	0.1520	0.1515																																													
14	***Xylosciara lignicola*** CAN BIOUG04314-A02	0.1260	0.1297	0.1462	0.1871	0.1617	0.1306	0.1540	0.1711	0.1695	0.1415	0.1462	0.1364	0.1586																																											
15	***Pseudolycoriella aotearoa*** NZL SDEI-Dipt-0000997	0.1824	0.1756	0.1641	0.2168	0.1687	0.1793	0.1884	0.1824	0.1960	0.1869	0.1869	0.1818	0.1778	0.1866																																										
16	***Pseudolycoriella bispina*** NZL SDEI-Dipt-0001522	0.1869	0.1883	0.1824	0.2203	0.1702	0.1839	0.1884	0.1884	0.1960	0.1854	0.1869	0.1717	0.1854	0.1851	0.1520																																									
17	***Pseudolycoriella bruckii*** GER BIOUG42711-H03	0.1969	0.1714	0.1771	0.2080	0.1939	0.1802	0.1710	0.1908	0.2122	0.1878	0.1710	0.1754	0.1847	0.1763	0.1802	0.1725																																								
18	***Pseudolycoriella brunnea*** GER BIOUG55426-H05	0.1677	0.1540	0.1646	0.2098	0.1738	0.1692	0.1754	0.1877	0.1923	0.1815	0.1723	0.1535	0.1708	0.1622	0.1677	0.1785	0.1738																																							
19	***Pseudolycoriella cavatica*** NZL SDEI-Dipt-0001411	0.1398	0.1440	0.1520	0.1888	0.1626	0.1489	0.1672	0.1657	0.1717	0.1565	0.1413	0.1481	0.1550	0.1462	0.1687	0.1550	0.1695	0.1646																																						
20	***Pseudolycoriella compacta*** GER BIOUG16613-B12	0.1550	0.1535	0.1581	0.1871	0.1884	0.1748	0.1793	0.1854	0.1809	0.1748	0.1778	0.1667	0.1778	0.1555	0.1763	0.1778	0.1679	0.1769	0.1337																																					
21	***Pseudolycoriella dagae*** NZL SDEI-Dipt-0000655	0.1824	0.1725	0.1809	0.2115	0.1869	0.1839	0.1884	0.1945	0.1991	0.1626	0.1884	0.1633	0.1657	0.1742	0.1687	0.1717	0.1649	0.1754	0.1611	0.1702																																				
22	***Pseudolycoriella frederickedwardsi*** NZL SDEI-Dipt-0000772	0.1368	0.1392	0.1337	0.1853	0.1702	0.1398	0.1565	0.1550	0.1581	0.1520	0.1459	0.1313	0.1489	0.1415	0.1717	0.1763	0.1695	0.1646	0.1383	0.1398	0.1687																																			
23	***Pseudolycoriella gonotegmenta*** NZL SDEI-Dipt-0000977	0.1461	0.1395	0.1476	0.1804	0.1674	0.1537	0.1705	0.1613	0.1750	0.1553	0.1492	0.1450	0.1537	0.1542	0.1750	0.1720	0.1682	0.1680	0.1400	0.1461	0.1598	0.0411																																				
24	***Pseudolycoriella hartmanni*** NOR TRD-Sci055	0.1429	0.1329	0.1489	0.1906	0.1672	0.1353	0.1626	0.1641	0.1657	0.1459	0.1489	0.1515	0.1429	0.1353	0.1763	0.1733	0.1573	0.1385	0.1368	0.1520	0.1702	0.1277	0.1370																																			
25	***Pseudolycoriella hauta*** NZL SDEI-Dipt-0000506	0.1626	0.1566	0.1960	0.2168	0.1763	0.1565	0.1869	0.1900	0.1809	0.1672	0.1733	0.1566	0.1778	0.1680	0.1884	0.1611	0.1817	0.1508	0.1398	0.1733	0.1337	0.1581	0.1537	0.1505																																		
26	***Pseudolycoriella horribilis*** KOR 74	0.1596	0.1693	0.1763	0.2133	0.1657	0.1550	0.1626	0.1793	0.1854	0.1733	0.1581	0.1279	0.1641	0.1633	0.1611	0.1520	0.1588	0.1323	0.1489	0.1581	0.1505	0.1398	0.1522	0.1520	0.1520																																	
27	***Pseudolycoriella huttoni*** NZL SDEI-Dipt-0000621	0.1489	0.1440	0.1459	0.1783	0.1763	0.1489	0.1657	0.1702	0.1687	0.1581	0.1489	0.1431	0.1505	0.1477	0.1733	0.1687	0.1679	0.1646	0.1398	0.1429	0.1687	0.0334	0.0304	0.1353	0.1550	0.1489																																
28	***Pseudolycoriella hygida*** ARG BIOUG23277-A10	0.1414	0.1549	0.1582	0.1783	0.1970	0.1498	0.1717	0.1751	0.1734	0.1650	0.1414	0.1573	0.1785	0.1448	0.1751	0.1751	0.1717	0.1667	0.1532	0.1700	0.1869	0.1532	0.1551	0.1414	0.1616	0.1684	0.1532																																
29	***Pseudolycoriella hygida*** ARG BIOUG23471-G01	0.1429	0.1519	0.1550	0.1783	0.1960	0.1459	0.1672	0.1778	0.1702	0.1596	0.1383	0.1566	0.1763	0.1431	0.1778	0.1748	0.1664	0.1662	0.1535	0.1702	0.1809	0.1520	0.1537	0.1413	0.1596	0.1687	0.1535	0.0000																															
30	***Pseudolycoriella hygida*** ARG BIOUG23645-D09	0.1444	0.1535	0.1535	0.1783	0.1976	0.1474	0.1657	0.1778	0.1717	0.1611	0.1368	0.1566	0.1748	0.1431	0.1793	0.1763	0.1679	0.1646	0.1520	0.1717	0.1824	0.1535	0.1553	0.1398	0.1611	0.1702	0.1550	0.0000	0.0015																														
31	***Pseudolycoriella hygida*** BRA SDEI-Dipt-0002255	0.1508	0.1584	0.1622	0.1754	0.1927	0.1527	0.1718	0.1698	0.1794	0.1756	0.1469	0.1587	0.1813	0.1450	0.1756	0.1737	0.1705	0.1552	0.1546	0.1679	0.1908	0.1565	0.1644	0.1355	0.1603	0.1698	0.1546	0.0039	0.0038	0.0038																														
32	***Pseudolycoriella hygida*** BRA SDEI-Dipt-0002256	0.1459	0.1503	0.1535	0.1748	0.1991	0.1489	0.1672	0.1748	0.1702	0.1596	0.1337	0.1566	0.1717	0.1415	0.1809	0.1778	0.1664	0.1631	0.1520	0.1702	0.1839	0.1505	0.1568	0.1383	0.1626	0.1687	0.1520	0.0034	0.0046	0.0030	0.0000																														
33	***Pseudolycoriella japonensis*** NOR bf-sci-00938	0.1717	0.1630	0.1748	0.2185	0.1809	0.1854	0.2097	0.1960	0.2188	0.1824	0.1839	0.1785	0.1641	0.1820	0.1687	0.1869	0.1832	0.1800	0.1763	0.1748	0.1793	0.1672	0.1674	0.1717	0.1915	0.1748	0.1672	0.1768	0.1778	0.1763	0.1737	0.1763																													
34	***Pseudolycoriella jaschhofi*** NZL SDEI-Dipt-0001511	0.1635	0.1640	0.1380	0.1970	0.2059	0.1699	0.1826	0.1699	0.1826	0.1741	0.1529	0.1691	0.1741	0.1645	0.1720	0.2038	0.1894	0.1763	0.1380	0.1592	0.1953	0.0934	0.1125	0.1465	0.1975	0.1699	0.0977	0.1775	0.1762	0.1741	0.1899	0.1699	0.2059																												
35	***Pseudolycoriella jejuna*** NZL SDEI-Dipt-0000603	0.1921	0.1730	0.1860	0.2207	0.1799	0.1936	0.1814	0.1966	0.2027	0.1890	0.1753	0.1686	0.1753	0.1841	0.1738	0.1814	0.1853	0.1759	0.1692	0.1799	0.0960	0.1692	0.1710	0.1677	0.1433	0.1616	0.1723	0.1855	0.1829	0.1814	0.1836	0.1799	0.1890	0.1940																											
36	***Pseudolycoriella jejunella*** NZL SDEI-Dipt-0000935	0.1824	0.1614	0.1930	0.2133	0.1884	0.1884	0.1809	0.2021	0.2021	0.1778	0.1839	0.1700	0.1657	0.1882	0.1717	0.1520	0.1740	0.1615	0.1565	0.1793	0.1383	0.1839	0.1796	0.1763	0.1155	0.1565	0.1778	0.1785	0.1763	0.1778	0.1813	0.1793	0.1793	0.2038	0.1555																										
37	***Pseudolycoriella kaikoura*** NZL SDEI-Dipt-0000500	0.1748	0.1725	0.1900	0.1976	0.1763	0.1854	0.1839	0.1793	0.1809	0.1915	0.1641	0.1599	0.1641	0.1835	0.1581	0.1535	0.1832	0.1677	0.1565	0.1672	0.1565	0.1672	0.1568	0.1733	0.1489	0.1550	0.1581	0.1667	0.1672	0.1687	0.1718	0.1687	0.1839	0.1805	0.1585	0.1611																									
38	***Pseudolycoriella macrotegmenta*** NZL SDEI-Dipt-0000757	0.1413	0.1503	0.1489	0.1871	0.1657	0.1535	0.1702	0.1626	0.1702	0.1611	0.1459	0.1498	0.1505	0.1524	0.1763	0.1733	0.1756	0.1646	0.1368	0.1489	0.1778	0.0334	0.0381	0.1337	0.1596	0.1474	0.0334	0.1566	0.1565	0.1581	0.1584	0.1550	0.1687	0.1019	0.1784	0.1854	0.1641																								
39	***Pseudolycoriella maddisoni*** NZL SDEI-Dipt-0000664	0.1611	0.1440	0.1657	0.1941	0.1748	0.1657	0.1672	0.1854	0.1641	0.1474	0.1717	0.1481	0.1565	0.1680	0.1672	0.1520	0.1756	0.1554	0.1413	0.1657	0.1170	0.1505	0.1446	0.1505	0.0912	0.1489	0.1520	0.1532	0.1535	0.1520	0.1565	0.1535	0.1733	0.1911	0.1296	0.1079	0.1429	0.1626																							
40	***Pseudolycoriella mahanga*** NZL SDEI-Dipt-0000595	0.1702	0.1725	0.1824	0.2063	0.1778	0.1733	0.1748	0.1793	0.1884	0.1763	0.1733	0.1717	0.1657	0.1820	0.1429	0.0851	0.1649	0.1646	0.1489	0.1581	0.1474	0.1581	0.1613	0.1672	0.1550	0.1429	0.1550	0.1667	0.1702	0.1687	0.1641	0.1672	0.1778	0.1805	0.1570	0.1489	0.1459	0.1565	0.1398																						
41	***Pseudolycoriella microteniuni*** SEY SDEI-Dipt-0002109	0.1402	0.1571	0.1402	0.1965	0.1905	0.1433	0.1845	0.1738	0.1768	0.1509	0.1494	0.1486	0.1723	0.1622	0.1723	0.1753	0.1776	0.1512	0.1372	0.1509	0.1585	0.1402	0.1481	0.1463	0.1540	0.1509	0.1418	0.1453	0.1509	0.1524	0.1475	0.1509	0.1784	0.1404	0.1774	0.1677	0.1631	0.1479	0.1555	0.1585																					
42	***Pseudolycoriella nodulosa*** NOR bf-sci-00732	0.1839	0.1772	0.1748	0.2290	0.1869	0.1824	0.2006	0.1991	0.2097	0.1748	0.1945	0.1801	0.1869	0.1742	0.1900	0.2021	0.1908	0.1631	0.1687	0.2006	0.1960	0.1748	0.1735	0.1687	0.1930	0.1763	0.1778	0.1717	0.1733	0.1717	0.1737	0.1733	0.1930	0.1762	0.2012	0.2097	0.1960	0.1717	0.1884	0.2067	0.1692																				
43	***Pseudolycoriella orite*** NZL SDEI-Dipt-0000863	0.1596	0.1566	0.1748	0.2133	0.1793	0.1611	0.1748	0.1869	0.1869	0.1687	0.1596	0.1785	0.1535	0.1664	0.1641	0.1520	0.1725	0.1646	0.1444	0.1657	0.1535	0.1489	0.1553	0.1565	0.1489	0.1398	0.1581	0.1616	0.1596	0.1581	0.1698	0.1565	0.1839	0.1592	0.1479	0.1596	0.1444	0.1581	0.1383	0.1261	0.1418	0.1824																			
44	***Pseudolycoriella paludum*** NOR bf-sci-00343	0.1216	0.1408	0.1550	0.1993	0.1611	0.1459	0.1520	0.1596	0.1641	0.1641	0.1596	0.1448	0.1626	0.1586	0.1748	0.1641	0.1603	0.1554	0.1337	0.1596	0.1611	0.1444	0.1476	0.1261	0.1474	0.1459	0.1444	0.1515	0.1535	0.1550	0.1546	0.1565	0.1793	0.1571	0.1616	0.1717	0.1535	0.1520	0.1520	0.1596	0.1341	0.1702	0.1337																		
45	***Pseudolycoriella plicitegmenta*** NZL SDEI-Dipt-0001153	0.1459	0.1408	0.1474	0.1801	0.1672	0.1535	0.1702	0.1611	0.1748	0.1550	0.1489	0.1448	0.1535	0.1540	0.1748	0.1733	0.1695	0.1677	0.1398	0.1459	0.1596	0.0410	0.0000	0.1368	0.1535	0.1520	0.0304	0.1549	0.1535	0.1550	0.1641	0.1565	0.1672	0.1125	0.1707	0.1793	0.1565	0.0380	0.1444	0.1626	0.1479	0.1733	0.1565	0.1474																		
46	***Pseudolycoriella porotaka*** NZL SDEI-Dipt-0001225	0.1748	0.1693	0.1687	0.1993	0.1839	0.1702	0.1748	0.1824	0.1915	0.1687	0.1733	0.1515	0.1672	0.1711	0.1641	0.1672	0.1695	0.1415	0.1459	0.1626	0.1474	0.1702	0.1750	0.1550	0.1459	0.1261	0.1702	0.1835	0.1824	0.1809	0.1794	0.1793	0.1687	0.1762	0.1616	0.1413	0.1535	0.1763	0.1413	0.1535	0.1524	0.1915	0.1444	0.1581	0.1748																	
47	***Pseudolycoriella puhihi*** NZL SDEI-Dipt-0001422	0.1793	0.1614	0.1793	0.2133	0.1839	0.1793	0.1733	0.1991	0.1976	0.1793	0.1611	0.1734	0.1581	0.1509	0.1611	0.1413	0.1740	0.1723	0.1520	0.1687	0.1626	0.1641	0.1659	0.1565	0.1565	0.1474	0.1626	0.1549	0.1550	0.1535	0.1565	0.1520	0.1809	0.1677	0.1692	0.1581	0.1474	0.1672	0.1535	0.1383	0.1555	0.1793	0.0881	0.1535	0.1672	0.1474																
48	***Pseudolycoriella raki*** NZL SDEI-Dipt-0000834	0.1746	0.1722	0.1730	0.2116	0.1761	0.1792	0.1945	0.1945	0.1899	0.1761	0.1807	0.1579	0.1761	0.1646	0.1593	0.1654	0.1923	0.1628	0.1516	0.1669	0.1041	0.1593	0.1656	0.1562	0.1286	0.1501	0.1685	0.1868	0.1838	0.1822	0.1869	0.1807	0.1730	0.1674	0.1229	0.1394	0.1562	0.1639	0.1149	0.1547	0.1551	0.2037	0.1485	0.1685	0.1654	0.1317	0.1577															
49	***Pseudolycoriella robustotegmenta*** NZL SDEI-Dipt-0001371	0.1489	0.1440	0.1505	0.1836	0.1702	0.1565	0.1717	0.1641	0.1748	0.1550	0.1535	0.1481	0.1565	0.1571	0.1763	0.1717	0.1679	0.1723	0.1444	0.1474	0.1596	0.0410	0.0061	0.1398	0.1535	0.1550	0.0304	0.1582	0.1565	0.1581	0.1679	0.1596	0.1641	0.1083	0.1738	0.1763	0.1611	0.0380	0.1459	0.1611	0.1524	0.1763	0.1596	0.1505	0.0061	0.1748	0.1702	0.1669														
50	***Pseudolycoriella subbruckii*** GER BIOUG07408-E12	0.1292	0.1329	0.1413	0.1923	0.1596	0.1398	0.1550	0.1596	0.1596	0.1565	0.1383	0.1481	0.1489	0.1337	0.1687	0.1626	0.1771	0.1400	0.1201	0.1383	0.1702	0.1383	0.1492	0.1140	0.1444	0.1444	0.1368	0.1414	0.1444	0.1429	0.1412	0.1398	0.1717	0.1507	0.1646	0.1657	0.1398	0.1459	0.1459	0.1489	0.1357	0.1672	0.1398	0.0638	0.1489	0.1505	0.1429	0.1593	0.1520													
51	***Pseudolycoriella submonticula*** GER BIOUG05458-H11	0.1626	0.1630	0.1809	0.2325	0.1793	0.1672	0.1869	0.2006	0.1915	0.1748	0.1945	0.1751	0.1854	0.1617	0.1809	0.2036	0.1863	0.1554	0.1839	0.1778	0.1915	0.1641	0.1583	0.1368	0.1733	0.1778	0.1596	0.1751	0.1778	0.1763	0.1679	0.1748	0.1626	0.1890	0.1905	0.1991	0.1809	0.1687	0.1809	0.1869	0.1784	0.1809	0.1778	0.1611	0.1596	0.1748	0.1778	0.1838	0.1626	0.1505												
52	***Pseudolycoriella subtilitegmenta*** NZL SDEI-Dipt-0001151	0.1474	0.1424	0.1489	0.1818	0.1687	0.1550	0.1717	0.1626	0.1763	0.1565	0.1505	0.1465	0.1550	0.1555	0.1763	0.1717	0.1710	0.1692	0.1413	0.1474	0.1611	0.0426	0.0015	0.1383	0.1550	0.1535	0.0319	0.1549	0.1535	0.1550	0.1641	0.1565	0.1687	0.1146	0.1723	0.1809	0.1581	0.0395	0.1459	0.1641	0.1494	0.1748	0.1581	0.1489	0.0015	0.1763	0.1687	0.1669	0.0076	0.1505	0.1611											
53	***Pseudolycoriella sudhausi*** NZL SDEI-Dipt-0001170	0.1581	0.1661	0.1611	0.1993	0.1763	0.1672	0.1733	0.2021	0.1733	0.1641	0.1824	0.1465	0.1687	0.1586	0.1611	0.1596	0.1847	0.1369	0.1489	0.1489	0.1413	0.1505	0.1583	0.1429	0.1353	0.1459	0.1520	0.1582	0.1550	0.1565	0.1508	0.1581	0.1854	0.1699	0.1524	0.1353	0.1474	0.1550	0.1277	0.1535	0.1341	0.1824	0.1413	0.1429	0.1581	0.1049	0.1581	0.1317	0.1581	0.1353	0.1672	0.1596										
54	***Pseudolycoriella teo*** NZL SDEI-Dipt-0001528	0.1255	0.1176	0.1569	0.1352	0.1137	0.1451	0.1529	0.1216	0.1804	0.1490	0.1569	0.1535	0.1333	0.1373	0.1294	0.0784	0.1502	0.1542	0.1020	0.1176	0.1059	0.1412	0.1102	0.1255	0.0941	0.1098	0.1294	0.1265	0.1294	0.1294	0.1294	0.1294	0.1255	0.0735	0.1063	0.1098	0.0980	0.1294	0.0980	0.0980	0.1220	0.1725	0.0824	0.0980	0.1137	0.1294	0.0863	0.1181	0.1216	0.1020	0.1529	0.1137	0.1216									
55	***Pseudolycoriella tewaipounamu*** NZL SDEI-Dipt-0000752	0.1687	0.1503	0.1839	0.1941	0.1960	0.1626	0.1778	0.1854	0.1717	0.1641	0.1748	0.1481	0.1611	0.1680	0.1763	0.1626	0.1832	0.1508	0.1489	0.1581	0.1277	0.1474	0.1537	0.1535	0.0881	0.1444	0.1444	0.1549	0.1581	0.1565	0.1489	0.1550	0.1809	0.1890	0.1418	0.1155	0.1474	0.1626	0.0608	0.1565	0.1540	0.1854	0.1353	0.1474	0.1535	0.1337	0.1489	0.1240	0.1550	0.1444	0.1748	0.1550	0.1185	0.1059								
56	***Pseudolycoriella tonnoiri*** NZL SDEI-Dipt-0000597	0.1459	0.1424	0.1368	0.1871	0.1657	0.1535	0.1702	0.1565	0.1778	0.1565	0.1535	0.1465	0.1444	0.1524	0.1672	0.1702	0.1725	0.1662	0.1383	0.1550	0.1641	0.0365	0.0228	0.1337	0.1520	0.1459	0.0289	0.1599	0.1596	0.1611	0.1679	0.1611	0.1596	0.1062	0.1784	0.1702	0.1626	0.0304	0.1489	0.1581	0.1509	0.1778	0.1581	0.1505	0.0228	0.1657	0.1657	0.1623	0.0228	0.1489	0.1702	0.0243	0.1550	0.1098	0.1550							
57	***Pseudolycoriella tuakana*** NZL SDEI-Dipt-0001337	0.1809	0.1661	0.1793	0.1993	0.1763	0.1824	0.1839	0.1900	0.1960	0.1657	0.1793	0.1684	0.1672	0.1633	0.1702	0.1733	0.1725	0.1692	0.1611	0.1672	0.0593	0.1626	0.1644	0.1565	0.1261	0.1581	0.1657	0.1801	0.1748	0.1763	0.1775	0.1748	0.1809	0.1826	0.0808	0.1322	0.1596	0.1687	0.1185	0.1550	0.1662	0.1824	0.1520	0.1626	0.1641	0.1292	0.1611	0.0842	0.1641	0.1565	0.1763	0.1657	0.1337	0.1137	0.1185	0.1672						
58	***Pseudolycoriella villabonensis*** ARG BIOUG24905-H03	0.1550	0.1598	0.1535	0.2080	0.1809	0.1474	0.1778	0.1809	0.1809	0.1596	0.1596	0.1532	0.1581	0.1555	0.1733	0.1854	0.1710	0.1446	0.1520	0.1565	0.1581	0.1505	0.1553	0.1429	0.1520	0.1581	0.1535	0.1431	0.1429	0.1413	0.1508	0.1429	0.1733	0.1720	0.1723	0.1520	0.1657	0.1550	0.1459	0.1687	0.1433	0.1626	0.1489	0.1596	0.1550	0.1520	0.1565	0.1593	0.1550	0.1383	0.1763	0.1565	0.1444	0.1255	0.1429	0.1474	0.1611					
59	***Pseudolycoriella wernermohrigi*** NZL SDEI-Dipt-0000665	0.1383	0.1440	0.1413	0.1853	0.1657	0.1505	0.1611	0.1596	0.1626	0.1596	0.1413	0.1448	0.1429	0.1431	0.1611	0.1717	0.1649	0.1600	0.1292	0.1429	0.1717	0.0380	0.0457	0.1246	0.1565	0.1444	0.0410	0.1498	0.1520	0.1535	0.1527	0.1505	0.1687	0.0913	0.1677	0.1793	0.1611	0.0258	0.1535	0.1550	0.1463	0.1733	0.1489	0.1459	0.0456	0.1672	0.1626	0.1654	0.0456	0.1413	0.1596	0.0471	0.1550	0.1333	0.1565	0.0380	0.1611	0.1459				
60	***Pseudolycoriella whakahara*** NZL SDEI-Dipt-0001439	0.1718	0.1438	0.1815	0.2089	0.1880	0.1702	0.1880	0.1880	0.1880	0.1702	0.1783	0.1534	0.1702	0.1761	0.1734	0.1653	0.1981	0.1506	0.1426	0.1799	0.1280	0.1637	0.1621	0.1588	0.0940	0.1588	0.1653	0.1616	0.1637	0.1621	0.1553	0.1637	0.1815	0.1953	0.1415	0.1248	0.1491	0.1718	0.0697	0.1637	0.1561	0.1799	0.1426	0.1572	0.1621	0.1361	0.1540	0.1275	0.1637	0.1556	0.1767	0.1637	0.1264	0.1075	0.0405	0.1605	0.1199	0.1459	0.1621			
61	***Pseudolycoriella whena*** NZL SDEI-Dipt-0001336	0.1535	0.1693	0.1657	0.1906	0.1641	0.1657	0.1672	0.1945	0.1641	0.1596	0.1763	0.1380	0.1687	0.1586	0.1581	0.1444	0.1771	0.1369	0.1505	0.1474	0.1216	0.1474	0.1553	0.1413	0.1231	0.1353	0.1535	0.1582	0.1565	0.1581	0.1508	0.1565	0.1839	0.1571	0.1418	0.1353	0.1596	0.1550	0.1216	0.1444	0.1341	0.1900	0.1353	0.1413	0.1550	0.0988	0.1444	0.1118	0.1550	0.1353	0.1702	0.1565	0.0502	0.1020	0.1155	0.1581	0.1170	0.1520	0.1565	0.1216		
62	***Pseudolycoriella zealandica*** NZL SDEI-Dipt-0000623	0.1733	0.1598	0.1687	0.2080	0.1687	0.1900	0.1641	0.1854	0.2112	0.1869	0.1793	0.1919	0.1687	0.1866	0.1292	0.1733	0.1695	0.1662	0.1550	0.1687	0.1596	0.1657	0.1644	0.1733	0.1809	0.1581	0.1687	0.1835	0.1839	0.1824	0.1947	0.1839	0.1641	0.1783	0.1692	0.1611	0.1565	0.1702	0.1535	0.1520	0.1784	0.1778	0.1489	0.1596	0.1641	0.1657	0.1489	0.1685	0.1672	0.1626	0.1748	0.1657	0.1687	0.1098	0.1717	0.1626	0.1626	0.1489	0.1596	0.1637	0.1626	
